# Study protocol of an open-label **p**rospective phase II **u**mb**r**ella study of **p**recise ne**o**adjuvant therapy for patients with **s**tage II-IIIB resectabl**e** non-small cell lung cancer (**PURPOSE**)

**DOI:** 10.3389/fonc.2022.1052774

**Published:** 2022-12-15

**Authors:** Yiyang Wang, Haoran Zhai, Jiaming Wang, Teng Mao, Chunyu Ji, Feichao Bao, Zhitao Gu, Wentao Fang

**Affiliations:** Department of Thoracic Surgery, Shanghai Chest Hospital, Shanghai Jiao Tong University School of Medicine, Shanghai, China

**Keywords:** neoadjuvant therapy, non-small cell lung cancer, locally advanced stage, umbrella study, next-generation sequencing

## Abstract

**Background:**

The outcomes of locally advanced non-small cell lung cancer (LA-NSCLC) are unfavorable mainly due to a high risk of cancer recurrence. Only around 5% of patients can benefit from perioperative chemotherapy which is the current standard treatment. Recently, promising results with neoadjuvant targeted and immune-therapy therapy have been seen. However, most clinical trials are looking for patients eligible for certain drugs, instead of seeking suitable treatments for certain patients. Therefore, it is necessary to look for more efficient perioperative therapies to increase resectability, reduce recurrence and improve prognosis.

**Methods/Design:**

The study is an open-label, prospective, phase II, umbrella trial, enrolling patients diagnosed with treatment-naïve potentially resectable Stage II-IIIB NSCLC. Next-generation sequencing (NGS) using a 68-gene panel is performed for biopsies of tumor tissues from eligible patients. Enrolled patients are then stratified into six independent cohorts based on the status of gene mutations and PD-L1 status in tumor tissues, that is, ①EGFR 19del group, ②EGFR 21 L858R group, ③EGFR rare mutation group, ④Other driver mutation group, ⑤Drive mutation-negative group with PD-L1≥1%, ⑥Drive mutation-negative group with PD-L1<1%. A Simon’s two-stage design is performed in each cohort independently and patients receive corresponding standard therapies accordingly. We aim to enroll 26 patients in each cohort and totally 156 patients will be enrolled. The primary endpoint is objective response rate (ORR). Secondary endpoints include oncological prognosis and perioperative outcomes. Exploratory endpoint is to investigate patient-specific minimal residual disease (MRD) in predicting treatment efficacy and oncological prognosis.

**Discussions:**

This is the first umbrella trial focusing on the safety and efficacy of precise neoadjuvant therapy for patients diagnosed with potentially resectable LA-NSCLC based on NGS results. The results of this trial would help improve overall treatment results in LA-NSCLC patients.

**Trial registration:**

Chinese Clinical Trial Registry. Trial registration number: ChiCTR2100053021.

**Advantages and limitations of this study:**

There is no neoadjuvant umbrella trial focusing on LA-NSCLCs. This is the first neoadjuvant umbrella trial, using a precise individualized approach and seeking suitable drugs for LA-NSCLC patients, with the aim to improve overall treatment outcomes.

**Clinical trial registration:**

https://www.chictr.org.cn/, identifier ChiCTR2100053021.

## Introduction

Lung cancer is the leading cause of cancer-related death around the world and in China (1, 2). Non-small cell lung cancer (NSCLC), as the most common pathological type, accounts for about 85% of all the lung cancers (3). Almost 20-25% patients already have locally advanced NSCLC (LA-NSCLC) (stage II-IIIB) upon diagnosis. Five-year overall survivals (OS) remain unsatisfactory even after surgical resection, ranging from only 26% to 60% (4, 5). Peri-operative chemotherapy has been the standard treatment for these patients (6, 7). However, there is only around 5% improvement of 5-year OS with post-operative adjuvant or pre-operative neoadjuvant chemotherapy (8, 9).

Recently, several early clinical trials have reported promising results to treat patients with epidermal growth factor receptor (EGFR) mutant LA-NSCLC with neoadjuvant targeted therapy. Erlotinib, a first-generation EGFR tyrosine kinase inhibitor (TKI) was used as neoadjuvant therapy in CTONG1103 and has been shown to improve the median progression-free survival from 11.4 months to 21.5 months among patients with EGFR mutant Stage IIIA (N2) NSCLC compared with chemotherapy (10). In the EASTERN trial, objective response rate (ORR) was 42.1% for patients with stage IIA (N2) NSCLC treated by erlotinib (11). In the NEOS study, an ORR of 71% and a R0 resection rate of 94% was achieved among patients with EGFR (+) LA-NSCLC treated with neoadjuvant Osimertinib, a third-generation TKI (12). Subsequently, clinical trials focusing on the third generation TKI as neoadjuvant therapy, such as NeoADAURA, are emerging. However, except for EGFR common mutations, other driver gene mutations have not be included in the design of these studies, such as EGFR rare mutations, anaplastic lymphoma kinase (ALK) rearrangement, mesenchymal-epithelial transition factor (MET) mutation, rearranged during transfection proto-oncogene (RET) rearrangement, and human epidermal growth factor receptor 2 (HER2) mutation.

Regarding patients without common driver gene mutations in the tumor, neoadjuvant immunotherapy combined with chemotherapy has been explored in some Phase II and III studies, such as NADIM, NEOSTAR and Checkmate 816 (13–15). In Checkmate 816, the pathological complete response (pCR) rate in patients treated with immunotherapy combined with chemotherapy was 24% compared with 2.2% in those receiving chemotherapy alone. The median event-free survival (EFS) between these two groups were 31.6 months versus 20.8 months, implying that neoadjuvant immunotherapy combined with chemotherapy could significantly improve of the outcomes in patients with LA-NSCLC (15). Nevertheless, it is still challenging to select patients who may benefit from immunotherapy, due to the lack of tumor biomarkers predicting the efficacy of immunotherapy.

Up till now, almost all clinical trials were designed to include eligible patients for certain treatment. With increasing knowledge in biomarkers and their association with potential response to treatment, it is now possible to select more appropriate therapy for a certain patient. Therefore, we hereby carry out the first umbrella study of precise neoadjuvant therapy for patients with potentially resectable LA-NSCLC (PURPOSE), to evaluate the ORR and long-term survival benefits based on next-generation sequencing (NGS).

## Methods

### Study setting

PURPOSE enrolls patients diagnosed with Stage II-IIIB potentially resectable NSCLC for precise neoadjuvant therapy followed by surgery, based on the NGS examination. This umbrella trial has been approved by the Institutional Review Board of Shanghai Chest Hospital (Approve date: October 26, 2021; approve number: IS21113) and has been registered at Chinese Clinical Trial Registry (ChiCTR) (Registration number: ChiCTR2100053021).

### Key inclusion criteria

(1) Patient age ≥18 years old and ≤75 years old;
(2) Histologically or cytologically confirmed NSCLC;
(3) Previously untreated NSCLC including local treatment (surgery or radiotherapy) and systemic anticancer therapy including chemotherapy, targeted therapy (TKI or monoclonal antibody), cell therapy, immunotherapy, Chinese medicine therapy and others;
(4) Stage II-IIIB N2 NSCLC should be confirmed according to the eighth TNM classification of the American Joint Committee on Cancer (AJCC) and evaluated by a multidisciplinary team (MDT) for the feasibility of surgical resection (Stage III is a diversified category and only stage IIIA and selected stage IIIB (N2) will be enrolled. In detail, we include patients with T1-2N2, T3-4N1, T4N0 of stage IIIA and selected patients with T3N2, T4N2 of stage IIIB. Patients with T4 invasion to organ, or bulky N2 disease are excluded). Mediastinoscopy or endobronchial ultrasonography biopsy of N2 disease suspected by computed tomography (CT) or positron emission tomography/computed tomography (PETCT) is strongly recommended;
(5)Sufficient tumor tissue (not cytology specimens) available for molecular marker analyses using NGS. The sufficient tumor tissue is defined as 15-20 slides of 5µm formalin fixed paraffin-embedded (FFPE) tumor tissues;
(6) At least one measurable lesion on spiral CT or PETCT according to the Response Evaluation Criteria in Solid Tumors version 1.1 (RECIST 1.1) (16);
(7) Eastern Cooperative Oncology Group (ECOG) Zubrod Performance Status (ZPS) 0-1 point;
(8) Patients should be able to tolerate neoadjuvant therapy and surgery, with forced expiratory volume in the first second (FEV1) ≥1.2L before surgery or predicted post-operative FEV1 ≥800ml;
(9) Good hematopoietic function, which is defined as absolute neutrophil count ≥1.5*10^9^/L, platelet count ≥100*10^9^/L, hemoglobin ≥9.0 g/dL;
(10) Good liver function, which is defined as serum total bilirubin ≤1.5 times of upper limits of normal (ULN), aspartate transaminase (AST) and alanine transaminase (ALT) ≤2.5 times of ULN;
(11) Good renal function, which is defined as serum creatinine ≤1.25 times of ULN or creatinine clearance rate (CCr) ≥60ml/min (Cockcroft-Gault), the proteinuria of urine routine test (URT) <2+;
(12) Good coagulation function, which is defined as the International normalized ration (INR) of prothrombin time (PT) ≤1.5ULN, activated partial thromboplastin time (APTT) ≤1.5ULN for patients not receiving anticoagulant therapy;
(13) Serum pregnancy test should be tested negative for women of childbearing age within 7 days before the first usage of investigational product. Contraception measures should be performed including intrauterine device, contraceptive pill or condom, or sterilization operation, for women of childbearing age and for male participants with female partner of childbearing age during clinical trial interval and within 90 days of last usage of investigational product;
(14) Life expectancy >6 months;
(15) Candidates are voluntary to participate in this study and the written informed consents should be signed (**Table 1**);

**Table 1:** Patient inclusion and exclusion criteria.

**Key inclusion criteria**
(1) Age≥18 years old and ≤75 years old; (2) Histologically or cytologically confirmed NSCLC; (3) Previously untreated NSCLC including local treatment (surgery or radiotherapy) and systemic anticancer therapy including chemotherapy, targeted therapy (TKI or monoclonal antibody), cell therapy, immunotherapy, Chinese medicine therapy and others; (4) Stage II-IIIB (N2) NSCLC should be confirmed according to the 8^th^ TNM classification of AJCC and evaluated by MDT for the feasibility of surgical resection. Mediastinoscopy or endobronchial ultrasonography biopsy of N2 disease suspected by CT or PETCT is strongly recommended; (5) Sufficient tumor tissue (not cytology specimens) available for NGS; (6) At least one measurable lesion on spiral CT or PETCT according to the RECIST 1.1; (7) ECOG ZPS 0-1 point; (8) Patients should be able to tolerate neoadjuvant therapy and surgery, with FEV1≥1.2L before surgery or predicted post-operative FEV1≥800ml; (9) Good hematopoietic function, which is defined as absolute neutrophil count≥1.5*109/L, platelet count≥100*109/L, hemoglobin≥9.0 g/dL; (10) Good liver function, which is defined as serum total bilirubin ≤ 1.5 times of ULN, aspartate transaminase and alanine transaminase ≤ 2.5 times of ULN; (11) Good renal function, which is defined as serum creatinine ≤ 1.25 times of ULN or creatinine clearance rate≥60ml/min (Cockcroft-Gault), the proteinuria of urine routine test<2+; (12) Good coagulation function, which is defined as INR of prothrombin time ≤ 1.5ULN, activated partial thromboplastin time ≤ 1.5ULN for patients not receiving anticoagulant therapy; (13) Serum pregnancy test should be performed for women of child bearing potential within 7 days before the first time usage of investigational product and the result of pregnancy test is negative. Contraception measures should be performed including intrauterine device, contraceptive pill or condom, or sterilization operation, for women of child bearing potential and male participants with female partner of child bearing potential during clinical trial interval and within 90 days of last usage of investigational product; (14) Life expectancy>6 months; (15) Candidates are voluntary to join this study and the informed consents should be signed;
**Key exclusion criteria**
(1) T4 stage including invasion to aorta, esophagus and/or heart, and/or bulky N2 disease; (2) Patients who cannot tolerate standard anatomical resection; (3) Diagnosed as SCLC or mixed type SCLC; (4) Patients with previous malignancies other than NSCLC within 5 years; (5) Unstable systemic diseases, including active infection, uncontrolled hypertension (Systolic blood pressure≥140mmHg or diastolic blood pressure≥90 mmHg), unstable angina pectoris, angina attacks within 3 months, liver or kidney diseases or metabolism disorder required medicine treatment; (6) Cardiac disease as following: ① Obvious arrhythmia with clinical significance which is not suitable for this trial, including complete left bundle branch block beat, second degree atrioventricular block, etc; ② Electrocardiogram: QTc interval ≥450ms (male), QTc interval ≥470ms (female); ③ NYHA ≥Grade 3 or LVEF <50% by ultrasonic cardiogram; ④ Myocardial infarction happened within one year before selection; (7) Non-infectious pneumonia needed glucocorticoid treatment within one year before neoadjuvant therapy or interstitial lung disease at present; (8) HIV infection;(9) Active Hepatitis B patient without treatment; (10) Active Hepatitis C patient; (11) Known drug allergies; (12) Major operation or severe damage with two months before the first neoadjuvant treatment; (13) Hemorrhage with clinical significance or obvious hemorrhagic tendency with one month before the first neoadjuvant treatment; (14) pregnant and lactating women; (15) received organ or blood system transplantation; (16) patient not suitable for this trial decided by the researchers, including nervous or metabolic disorder, potential diseases or contraindications to research drugs or high risk of treatment-related complications; (17) patients will be excluded before receiving Camrelizumab plus chemotherapy, ① Active autoimmune diseases needed systemic therapy within two years before the first neoadjuvant treatment; ② Immune deficiency or systemic glucocorticoid treatment not related with oncological therapy within 7 days before the first neoadjuvant treatment or other suppression immunotherapies; ③ Severe infection (CTCAE ≥Grade 3) within 4 weeks before the first neoadjuvant treatment, including infectious complications needed in-hospital treatment, bacteremia, severe pneumonia, etc; ④ Vaccination (active vaccine/inactivated vaccine) within 30 days before the first neoadjuvant treatment;
**Patient withdrawal**
(1) SAE threatens the safety of patients; (2) Poor patient compliance; (3) Patient requires to withdrawal or terminate the study for personal reasons;

NSCLC, non-small cell lung cancer; TKI, tyrosine kinase inhibitor; AJCC, American Joint Committee on Cancer; MDT, multidisciplinary team; CT, computed tomography; PETCT, positron emission tomography/computed tomography; NGS, next generation sequencing; RECIST 1.1, Response Evaluation Criteria in Solid Tumors version 1.1; ECOG, Eastern Cooperative Oncology Group; ZPS, Zubrod Performance Status; FEV1, the forced expiratory volume in one second; ULN, upper limits of normal; INR, International normalized ration; SCLC, small cell lung cancer; NYHA, New York Heart Association; LVEF, left ventricular ejection fraction; HIV, Human immunodeficiency virus; CTCAE, the Common Terminology Criteria for Adverse Events; SAE, serious adverse events.

### Key exclusion criteria

(1) T4 stage including invasion to aorta, esophagus and/or heart, and/or bulky N2 disease;
(2) Patients who cannot tolerate a standard anatomical resection;
(3) Patients diagnosed with small cell lung cancer (SCLC) or mixed type of SCLC;
(4) Patients with previous malignancies other than NSCLC within 5 years;
(5) Unstable systemic diseases, including active infection, uncontrolled hypertension (Systolic blood pressure ≥140mmHg or diastolic blood pressure ≥90mmHg), unstable angina pectoris, angina attacks within 3 months, liver or kidney diseases or metabolism disorder required medicine treatment;
(6) Cardiac disease as following:
① Obvious arrhythmia with clinical significance, which is not suitable for this trial, including complete left bundle branch block beat, second degree atrioventricular block, etc.;
② Electrocardiogram: QTc interval ≥450ms (male), QTc interval ≥470ms (female);
③ New York Heart Association (NYHA) ≥Grade 3 or left ventricular ejection fraction (LVEF) <50% by ultrasonic cardiogram;
④ Myocardial infarction happened within one year before selection;
(7) Non-infectious pneumonia needed glucocorticoid treatment within one year before neoadjuvant therapy or interstitial lung disease at present;
(8) Human immunodeficiency virus (HIV) infection;
(9) Active Hepatitis B patient without treatment;
(10) Active Hepatitis C patient;
(11) Known drug allergies;
(12) Major operation or severe damage with two months before the first neoadjuvant treatment;
(13) Hemorrhage with clinical significance or obvious hemorrhagic tendency with one month before the first neoadjuvant treatment;
(14) Pregnant and lactating women;
(15) Received organ or blood system transplantation;
(16) Patient not suitable for this trial decided by the researchers, including nervous or metabolic disorder, potential diseases or contraindications to research drugs or high risk of treatment-related complications;
(17) Patients will be excluded before receiving Camrelizumab plus chemotherapy,
① Active autoimmune diseases needed systemic therapy within two years before the first neoadjuvant treatment;
② Immune deficiency or systemic glucocorticoid treatment not related with oncological therapy within 7 days before the first neoadjuvant treatment or other suppression immunotherapies;
③ Severe infection (Common Terminology Criteria for Adverse Events ≥Grade 3) within 4 weeks before the first neoadjuvant treatment, including infectious complications needed in-hospital treatment, bacteremia, severe pneumonia, etc;
④ Vaccination (active vaccine/inactivated vaccine) within 30 days before the first neoadjuvant treatment (**Table 1**);

### Patient withdrawal

During the entire study, enrolled candidates will be re-evaluated if any of the following criteria is met:

(1) Serious adverse events (SAE) threaten the safety of patients;
(2) Poor patient compliance;
(3) Patient requires to withdrawal or terminate the study for personal reasons (**Table 1**).

### Study design

**Figure 1:**
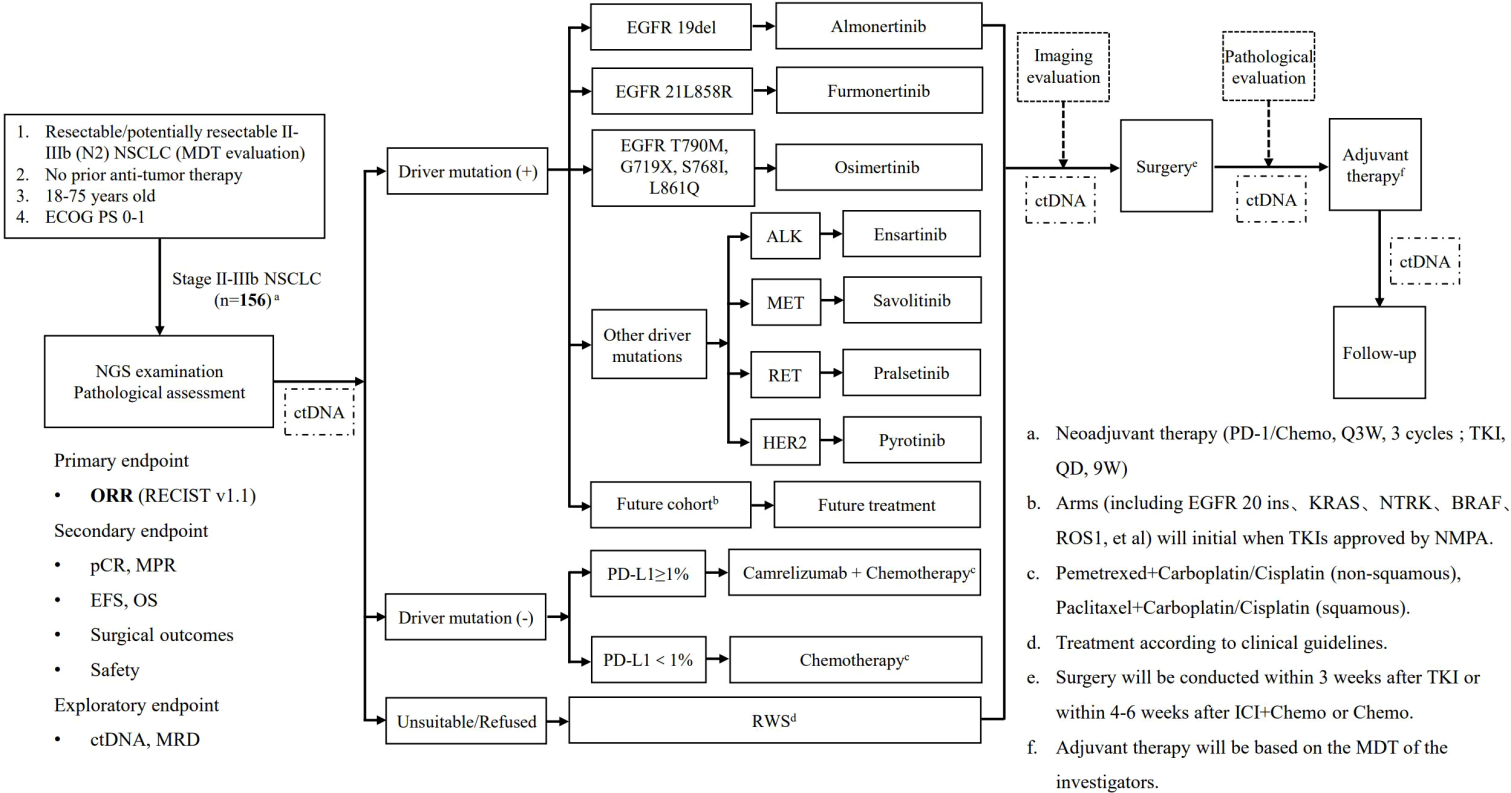
The study flowchart.

The design of this study is show in **Figure 1**. Histological examinations along with a 68-gene panel NGS (Burning Rock Biotech, Guangzhou, China) and programmed death-ligand 1 (PD-L1) immunohistochemical staining (Dako Monoclonal Mouse Anti-Human PD-L1, Clone 22C3) are performed for tumor biopsies (including percutaneous pulmonary biopsy, transbronchial or endobronchial ultrasound biopsy) from enrolled patients. A personalized minimum residual disease (MRD) examination is also conducted for pretreatment tumor biopsies and peripheral blood circulating tumor DNA (ctDNA) by Burning Rock Biotech (Guangzhou, China). Pre- and immediate post-operative ctDNA and ctDNA during follow-up intervals in peripheral blood samples are analyzed for exploratory research.

Based on the NGS and PD-L1 results, qualified patients are divided into six independent cohorts, that is, ①EGFR 19del group, ②EGFR 21 L858R group, ③EGFR rare mutation group, ④Other driver mutation group, ⑤Driver mutation-negative group with PD-L1≥1%, ⑥Driver mutation-negative group with PD-L1<1%. Patients in each cohort receive corresponsive neoadjuvant therapy accordingly. Patients enrolled in the EGFR 19del group receive Almonertinib (110mg, QD; Q3W, 3 cycles). Patients in the EGFR 21 L858R group receive Furmonertinib (80mg, QD; Q3W, 3 cycles). Patients in the EGFR rare mutation group (T790M/G719X/S768I/L861Q) receive Osimertinib (80mg, QD; Q3W, 3 cycles). In the group with other driver mutations such as ALK/MET/RET/HER2, Ensartinib (225mg, QD; Q3W, 3 cycles) for ALK+ tumors, Savolitinib (600mg, QD; Q3W, 3 cycles) for MET+ tumors, Pralsetinib (400mg, QD; Q3W, 3 cycles) for RET+ tumors, Pyrotinib (400mg, QD; Q3W, 3 cycles) for HER2+ (HER2 Exon 20 mutation) tumors. In addition, tumors with other gene mutations, such as EGFR 20 ins, kirsten ratsarcoma viral oncogene homolog (KRAS), neuro trophin receptor kinase (NTRK), v-raf murine sarcoma viral oncogene homolog B1 (BRAF) Other gene mutations, such as EGFR 20ins, KRAS, BRAF, NTRK, are not included in this study temporarily. Such patients will be enrolled into the real world group receiving standard treatment decided by their physicians. This study design allows opening up to additional cohorts so that patients with these gene mutations will be included in the future once corresponding drugs are approved by NMPA of China or showed confirmed efficacy in strictly designed clinical trialsPD-L1 status is tested in patients without driver gene mutation. Patients with PD-L1≥1% tumor receive Camrelizumab (200mg, Q3W) in combination with chemotherapy, and patients with PD-L1<1% tumors receive chemotherapy alone. Pemetrexed (500mg/m^2^, Q3W) combined with carboplatin (AUC=5) or cisplatin (75mg/m^2^, Q3W) is applied as neoadjuvant chemotherapy for non-squamous NSCLC, and paclitaxel (135~175mg/m^2^, Q3W) with carboplatin (AUC=5) or cisplatin (75mg/m^2^, Q3W) for squamous cell carcinoma. Eligible patients declining to join this PURPOSE trial will be enrolled into a real-world study cohort, and treatment is performed according to current clinical guidelines (**Figure 1**).

### Neoadjuvant treatment response evaluation

Tumor response to treatment is evaluated by imaging scans within two weeks after neoadjuvant therapy, according to RECIST version 1.1 (17). Examinations after neoadjuvant therapy include enhanced chest CT, brain magnetic resonance imaging (MRI) and PET-CT. Each patient enrolled will be fully evaluated and discussed the feasibility of receiving surgical resection by MDT team right after completing neoadjuvant response examinations.

### Thoracic surgery and pathological assessment

For patients with resectable LA-NSCLC, surgery is conducted within 3 weeks after TKI treatment and within 4-6 weeks after camrelizumab plus chemotherapy treatment or chemotherapy alone. Open thoracotomy or minimally invasive surgery are conducted according to the surgeons’ judgement. Anatomical resections including lobectomy, bilobectomy, sleeve lobectomy, pneumectomy combined with systemic lymph node dissection, are recommended. Detailed intraoperative and postoperative outcomes are recorded for the further assessment. Pathological evaluation is carried out after surgery, and pCR or major pathologic response (MPR) is cross examined by two experienced pathologists from the Department of Pathology, Shanghai Chest Hospital.

### Postoperative treatment

All patients who have received thoracic surgery and patients with unresctable tumors due to non-disease related progression continue to receive adjuvant therapy, according to the MDT discussions. Adjuvant therapy strategy is decided by the MDT according to the current treatment guidelines.

### Follow-up strategy

All patients enrolled is followed at our out-patient department. Chest CT scan, neck and abdominal ultrasound, serum tumor makers are performed every 6 months, and brain MRI every year, during the first three years. In the fourth and fifth years, chest CT scan, brain MRI, neck and abdominal ultrasound, serum tumor makers is performed every year. PETCT is recommended when local recurrence or distant metastasis is suspected. All the follow-up data and information of examinations and evaluations will be carefully recorded.

### Study endpoints

The primary endpoint of this study is objective response rate (ORR), defined as the proportion of patients with complete response (CR) and partial response (PR), to estimate the treatment efficacy according to RECIST 1.1.The secondary endpoints include pCR and MPR, oncological prognosis including event-free survival (EFS) and OS, surgical outcomes and adverse events associated with neoadjuvant treatment. pCR is defined as zero percentage of residual viable tumor cells in the primary tumor and dissected lymph nodes. MPR is defined as 10% residual viable tumor cells in the primary tumor. EFS is defined as the time from the beginning of neoadjuvant treatment to any progression of disease, including disease progression that cannot receive surgical operation, local recurrence or distant metastasis, death from any cause. OS is defined as the time from the beginning of neoadjuvant treatment to death from any cause. The safety of neoadjuvant therapies is evaluated by adverse events assessed by Common Terminology Criteria for Adverse Events version 5.0 (CTCAE 5.0) in all the treated patients.

Exploratory endpoint is to investigate the relationship between oncological prognosis and MRD. The detection and quantification of ctDNA for MRD assessment was investigated using brPROPHETTM, a personalized, tumor-informed ctDNA assay designed to track up to 50 top-ranked patient-specific somatic variants based on whole exome sequencing (WES) of the tumor tissue and matched white blood cells (WBCs) (Burning Rock Biotech, Guangzhou, China). The time points of MRD testing for each enrolled patient in this study are as following, ① the first time of ctDNA testing is right after tumor biopsy and before neoadjuvant therapy, ② the second time of ctDNA testing is within 3 days before surgical resection, ③ the third time of ctDNA testing is the first month after surgical resection, ④ the fourth time of ctDNA testing is the six month after surgical resection.

### Sample size

According to statistical estimation of sample size with an alternative hypothesis of ORR>60% and a null hypothesis of ORR<36%, α=5%, β=20%, each cohort will enroll 26 patients, with a total of 156 participants for the entire trial. The sample size was calculated by software NCSS PASS 2021 (LLC. Kaysville, Utah, USA).

Each clinical cohort will be carried out independently in a Simon’s mini-max two-stage design. In Simon’s mini-max stage one, if less than 3 out of 8 qualified patients had complete response/partial response (CR/PR), the related cohort will be terminated for the poor efficacy of neoadjuvant treatment. Otherwise, an additional 18 patients will be enrolled into stage two. Taking together, if less than 13 patients have CR/PR, the result of this group will be considered as a poor clinical outcome. If more than 13 qualified patients have CR/PR, the neoadjuvant therapy for this cohort will be considered as an efficacy treatment.

### Statistical analysis

In this clinical trial, we define several datasets as follows. Full analysis set (FAS) is defined as the patients who meet all the inclusion criteria and receive at least one cycle of neoadjuvant treatment according to the intent-to-treat (ITT) principles. Per protocol set (PPS) is defined as FAS patients who complete the whole schedule of treatment and follow-up, do not take prohibited medications and the case report forms are all completed without any missing data. Safety set (SS) is defined as all the enrolled patients receiving at least one dose of the neoadjuvant treatment for the assessment of drug safety analysis.

For the descriptive statistical analysis, the categorical variables are presented as number and percentage, compared through the Pearson χ2 or Fisher’s exact test. The continuous variables are presented as mean and standard deviation (SD), compared through the Students’ t test for the normally distributed continuous variables and the Mann–Whitney U-test for the non-normally distributed ones. For the survival analysis of prognostic outcomes, the survival curves of EFS, DFS and OS, along with related median survival and survival rate are plotted by the Kaplan-Meier methods. The 95% confidence interval (CI) of survival are analyzed by the Brookmeyer-Crowley method with the two-side Log-rank test.

For the safety analysis. The occurrence of adverse events (AE) are recorded and coded according to the Medical Dictionary for Regulatory Activities (MedDRA) (16). And the AEs are summarized by the System Organ Class (SOC), Preferred Term (PT), the correlation with study drugs and the severity of Common Terminology Criteria for Adverse Events version 5.0 (CTCAE 5.0) (18).

Two-sided p<0.05 is set as a statistically significance. All the statistical analysis are performed by using SAS (version 9.4, Cary, NC) and R software (version 4.1.1).

## Discussions

Neoadjuvant targeted therapy and neoadjuvant immunotherapy are promising approaches in the treatment of patients with LA-NSCLC, as were shown in several clinical trials including NEOS and Checkmate 816 (12, 15). However, the majority of clinical trials are designed to seek patients for a certain drug, rather than seek a appropriate drug for a certain patient. This limits the improvement of the survival benefits in the overall population diagnosed with LA-NSCLC. Therefore, it is imperative for clinicians to find a better way to implement tailored precise treatment for all patients.

In 2014, American Association for Cancer Research (AACR) has pointed out the ideas of “basket study” and “umbrella study”. Unlike basket study in which a given therapy is used for patients with any type of cancers carrying the same genetic mutation, umbrella study aims to explore the optimal treatment for patients with the same cancer type harboring different driver gene mutations (19). Considering that lung cancer consists of heterogeneous tumors carrying distinct targetable driver gene mutations such as KRAS, EGFR, ALK, umbrella study can be perfectly implemented for lung cancer studies to explore the efficacy of different targeted drugs for patients with lung cancer.

The Lung Cancer Master Protocol (Lung-MAP; S1400) promoted by the United States National Cancer Institute (NCI), was a typical umbrella trial, which was completely a biomarker-driven clinical trial aiming to provide better therapy strategies for patients with recurrent or metastatic squamous non-small-cell lung cancer. In this umbrella trial, patients with stage IV or recurrent squamous non-small-cell lung received assigned therapies depended on different biomarkers or population (20). The National Lung Metrix Trial (NLMT), which is so far the largest umbrella trial, aimed to explore the prognosis of patients with tumors carrying different driver gene mutations when receiving corresponsive targeted (21). However, NLMT only enrolled patients with metastatic lung cancer and only 40% of tumors from those patients were sent for genetic sequencing. More importantly, only 5% of these patients successfully received the matched treatment. Nevertheless, NLMT has offered a promising model using umbrella trial to improve the benefits of patients with lung cancer from precise treatment.

In the aspect of neoadjuvant umbrella study, the LCRF-LEADER screening trial (NCT04712877), promoted by the Lung Cancer Mutation Consortium (LCMC) and Thoracic Surgery Oncology Group (TSOG), is aimed to determinate the proportion of oncogenic drivers and to select the optimal neoadjuvant therapy for stage IA2-III lung cancer patients (22). However, LCRF-LEADER is actually a collaborative screening trial, and the study population is preferred patients with resectable non-squamous non-small cell lung cancer, but not the overall population. NAUTIKA1 study is a phase II umbrella trial focusing on the neoadjuvant therapy including ALK, ROS1, NTRK, BRAF V600E and PD-L1 positive in stage II-III NSCLC patients. Recently, the preliminary results of neoadjuvant targeted therapy for ALK+ NSCLC have been reported in WCLC 2022 (23). Totally 8 ALK+ NSCLC patients have been enrolled and 5 patients have completed neoadjuvant alectinib therapy and received surgical resection. The R0 rate was 100% with no severe complications, indicating the safety and feasibility of alectinib for the neoadjuvant therapy of stage II-III ALK-positive NSCLC patients. Different from NAUTIKA1 study focusing on the rare driver mutations such as ALK, ROS1, NTRK, BRAF V600E, our PURPOSE trial focuses on the entire population including not only common mutations (such as EGFR 19del and EGFR 21 L858R) and rare mutations (such as ALK, RET, MET, HER2), but also PD-L1 positive and negative patients. For other mutations not included in this trial right now such as EGFR 20ins, KRAS, BRAF, NTRK, et al, this study design allows opening up to additional cohorts so that patients with these gene mutations will be included in the future once corresponding drugs are approved by NMPA of China or showed confirmed efficacy in strictly designed clinical trials. For the selection of the third generation TKI (Furmonertinib, Almonertinib, Osimertinib) according to different EGFR mutation types, it was fully considered by PURPOSE trial group. According to FLAURA, AENEAS and FURLONG trials, the third generation TKIs (Osimertinib, Almonertinib, Furmonertinib) showed significantly better survival prognosis compared with the first generation TKIs (24–26). Considering the mPFS of Almonertinib was 19.3 months and the mPFS of Osimertinib was 18.9 months, we chose Almonertinib as our priority to EGFR 19del/21 L858R. And we have known that EGFR 19del and L858R were two different diseases (24). Previous studies have pointed out that the treatment efficacy of Furmonertinib was better than Almonertinib in the subgroup of L858R in patients with EGFR T790M+ NSCLC (27, 28). Therefore, we selected Furmonertinib as the neoadjuvant treatment in the group of EGFR 21L858R and Almonertinib in the group of EGFR 19del. According to a previous review, Osimertinib and Afatinib were all suggested as possible first-line treatment options for the major uncommon EGFR mutations including G719X/S768I/L861Q.Besides, Osimertinib was the only approved TKI for EGFR T790M-positive NSCLC patients. Therefore, we chose Osimertinib over Afatinib in the EGFR rare mutation group (T790M/G719X/S768I/L861Q) (29).

Hence, this PURPOSE trial, to our best knowledge. is the first real-sense umbrella study in overall population designed to explore the neoadjuvant therapy for LA-NSCLCs. Therefore, PURPOSE will enable us to optimize the strategy of precise individualized treatment for LA-NSCLC, and improve the overall treatment outcomes in these patients.

## Data availability statement

The datasets presented in this article are not readily available because the dataset will be restricted within the five years after the trial finished. Requests to access the datasets should be directed to YW, wangyiyang_sch@163.com.

## Ethics statement

This umbrella trial has been approved by the Institutional Review Board of Shanghai Chest Hospital (Approve date: October 26, 2021; approve number: IS21113) and has been registered at Chinese Clinical Trial Registry (ChiCTR) (Registration number: ChiCTR2100053021). The patients/participants provided their written informed consent to participate in this study.

## Author contributions

Concept Proposal: WF. Survey and Data Summary: YW and HZ. Data Collection, analysis and statistics: JW, TM, CJ, FB, ZG. Research Regulatory: WF. Writing – Draft: YW. Writing-Proofreading and Editing: YW and HZ. All authors contributed to the article and approved the submitted version.
